# Vitamin D Status among 2–18-Year-Old Romanian Pediatric Patients: A Single-Center Study

**DOI:** 10.3390/nu16142266

**Published:** 2024-07-14

**Authors:** Ioana Badiu Tișa, Anamaria Cozma-Petruț, Gabriel Samașca, Doina Miere, Lorena Filip, Roxana Banc, Oana Mîrza, Mihaela Iancu

**Affiliations:** 1 Department of Mother and Child Care, “Iuliu Hațieganu” University of Medicine and Pharmacy, 2-4 Câmpeni Street, 400217 Cluj-Napoca, Romania; ioana.badiu@umfcluj.ro; 2 Department of Bromatology, Hygiene, Nutrition, “Iuliu Hațieganu” University of Medicine and Pharmacy, 6 Pasteur Street, 400349 Cluj-Napoca, Romania; dmiere@umfcluj.ro (D.M.); lfilip@umfcluj.ro (L.F.); roxana.banc@umfcluj.ro (R.B.); oana.stanciu@umfcluj.ro (O.M.); 3 Department of Functional Biosciences, Immunology and Allergology, “Iuliu Hațieganu” University of Medicine and Pharmacy, 19-21 Croitorilor Street, 400162 Cluj-Napoca, Romania; gabriel.samasca@gmail.com; 4 Academy of Romanian Scientists (AOSR), 3 Ilfov Street, 050044 Bucharest, Romania; 5 Department of Medical Informatics and Biostatistics, “Iuliu Hațieganu” University of Medicine and Pharmacy, 6 Pasteur Street, 400349 Cluj-Napoca, Romania; miancu@umfcluj.ro

**Keywords:** vitamin D, 25(OH)D, deficiency, insufficiency, children, sex, age, season, Romania

## Abstract

An adequate vitamin D level is essential for optimal bone mass formation during growth. The present study aimed to assess (i) the sex-specific, age-specific, and potential seasonal (spring, summer, winter) influences on the pediatric circulating levels of 25-hydroxyvitamin D (25(OH)D); (ii) determine the frequency of pediatric patients with vitamin D deficiency (VDD) or insufficiency (VDI); and (iii) quantify the association between age category, sex, and season types and susceptibility to VDD and VDI, respectively. Laboratory data were collected on serum 25(OH)D levels in children aged between 2 and 18 years (n = 1674) who underwent blood sampling following admission to a university pediatric hospital in Cluj-Napoca (Romania) between January and June 2023. VDD (<20 ng/mL) was observed in 27% of pediatric patients. Among toddlers and preschoolers (2–5 years), VDD was 11%, while it was 33% among school-aged children (6–11 years) and 39% among adolescents (12–18 years). We found a significant difference in the frequencies of vitamin D status between females and males (*p* = 0.006). Also, we found significant associations of vitamin D status with age categories (*p* < 0.0001) and seasonal variations (*p* = 0.03). After adjusting for season of blood collection, the multinomial logistic regression model showed that children aged 6–11 years old (adjusted OR = 7, 95% CI: (4.9, 9.4)), children aged 12–18 years old (adjusted OR = 14, 95% CI: (9.3, 19.6)), and females (adjusted OR = 1.43, 95% CI: (1.10, 1.86)) were significantly associated with higher odds of VDD. In conclusion, the study revealed a significant difference in the frequency of VDD and VDI among pediatric patients older than six years, with a significant difference according to sex and season, being more pronounced among girls and during the winter and spring seasons.

## 1. Introduction

Knowledge of the relationship between vitamin D status and health in children has increased significantly in recent decades. Vitamin D is a fat-soluble prehormone essential for calcium and phosphorus homeostasis and healthy bone metabolism. In addition, vitamin D has an important role in regulating the immune system and exhibits anti-inflammatory and antioxidant actions [1,2,3,4,5].

Although vitamin D performs key biological functions, vitamin D deficiency (VDD) and vitamin D insufficiency (VDI) currently represent a public health issue, with an increasing prevalence globally [6]. VDD is usually defined as a condition where serum 25(OH)D levels are below 50 nmol/L or 20 ng/mL. Furthermore, VDI is often defined as serum 25(OH)D levels between 21 and 29 ng/mL (52.5–72.5 nmol/L) [7].

VDD can lead to bone hypomineralization disorders [8,9,10]. For instance, VDD during childhood has been associated with adverse outcomes like growth failure and rickets [11]. Moreover, VDD is widespread among children living in temperate climates and has been reported to be associated with obesity and early activation of the hypothalamic–pituitary–gonadal axis [6,12]. VDD is also associated with many other acute and chronic extraskeletal diseases, including autoimmune diseases, cancer, type 2 diabetes, cardiovascular diseases, and depression [3,4,13]. Infectious disorders, including upper respiratory tract infections and severe coronavirus infections (COVID-19), are also linked to VDD [14,15,16]. Severe VDD, with a concentration of 25(OH)D below 30 nmol/L or 12 ng/mL, raises the risk of infections, many other illnesses, and even death and should be avoided at all costs. Therefore, a level of 25(OH)D >50 nmol/L or 20 ng/mL is the main treatment objective, although some evidence points to the advantages of a higher threshold [17,18,19].

Since vitamin D can be obtained through cutaneous synthesis or from dietary sources, most individuals in industrialized countries have a combination of risk factors leading to VDD. Indeed, VDD most commonly occurs in childhood due to a combination of inadequate cutaneous synthesis and poor dietary intake. Cutaneous synthesis can be limited due to the ineffectiveness of sunlight in stimulating vitamin D synthesis during winter periods and decreased cutaneous synthesis due to increased skin pigmentation. It is now recognized that maintaining a sufficient vitamin D level is essential during growth periods for optimal bone mass formation in childhood and later in preventing osteoporosis in adults [20,21,22].

The status of vitamin D has been thoroughly assessed in the past few decades in different populations across the globe, spanning various age groups [11]. Recent reports indicate that VDD is common in the population in Europe [23]. Concerning Romania, located in southeastern Europe, between 44° N and 48° N latitude, there are some data available regarding the vitamin D status in the adult population of this country [24,25,26,27]. For instance, one recent study reported a prevalence of VDD of 24.8% in an adult population sample randomly selected to represent the Romanian population aged between 25 and 64 years [26]. Nevertheless, there are only limited data on vitamin D status in the pediatric population of Romania [24,28]. In this context, the current study assessed the vitamin D status of a sample of children aged 2–18 years admitted to a pediatric hospital in Cluj-Napoca, Romania, under the following objectives: (i) to test the effect of age category, sex, and season types (spring, summer, winter) on serum 25(OH)D values; (ii) to estimate the frequency of VDD in the studied sample of pediatric patients; and (iii) to quantify the influence of age category, sex, and season types on risk of VDD and VDI, respectively.

## 2. Materials and Methods

The serum concentration of 25(OH)D was evaluated, and the thresholds for VDD and VDI were established at serum 25(OH)D concentrations of <20 ng/mL and 21–29 ng/mL, respectively, with sufficient vitamin D levels identified at serum concentrations >30 ng/mL [29,30,31,32].

### 2.1. Data Collection and Participants

Data on vitamin D status were retrospectively collected from the laboratory records of the Emergency Clinical Hospital for Children Cluj-Napoca, a university pediatric hospital located in the northwestern region of Romania. Except for date of birth and sex, no personal data were included in the dataset. Data were retrieved on children (aged 2–18 years) who were admitted to the pediatric hospital for specialized investigations between January and June 2023 (n = 1674) and from whom fasting venous blood samples were collected to determine the serum 25(OH)D concentration. Considering that the study was retrospective and anonymous, individual information on vitamin D supplementation, dietary intake, and sun exposure level was not available.

Children with underlying chronic diseases, such as absorption disorders, genetic syndromes, or skeletal malformations, were not included in the study.

Basic demographic characteristics of the participants, including sex, age, and data regarding season, were collected. Age was stratified into three groups: toddler and preschool age (2–5 years), school age (6–11 years), and adolescence (12–18 years). Seasons were categorized into three groups: winter (January, February), spring (March, April), and summer (May, June).

### 2.2. Sample Collection and Analysis

To assess vitamin D levels, the concentration of 25(OH)D, the primary circulating form of vitamin D, was used.

Paraclinical tests were conducted using the chemiluminescent immunoassay method on the Mindray SAL 6000 system (BS800 + CL 2000) provided by Mindray Medical International Limited, Shenzhen, Guangdong, China. For the immunological tests, fasting venous blood samples were collected into a sterile 4 mL vacutainer without anticoagulant and centrifuged for 10 min at 2000× *g* to separate the serum. The analysis required 40 µL of serum to determine vitamin D level.

The manufacturer provided the inclusion levels for vitamin D as follows: <20 ng/mL indicating deficiency, 21–29 ng/mL indicating insufficiency, and >30 ng/mL indicating sufficiency, respectively.

### 2.3. Statistical Analysis

Demographic variables (age, sex, season of blood sample collection) were summarized by arithmetic mean with sample standard deviation (SD) or frequencies (%). The 25(OH)D serum concentration had departures from the Gaussian distribution, and it was summarized by a median with an interquartile interval IQR = [25th percentile; 75th percentile]. Evaluation of univariate Gaussian distribution was performed using several methods, such as descriptive statistics, quantile–quantile (Q-Q) plot, and the Shapiro–Wilk test, with Holm correction for multiple comparisons.

Variations in the 25(OH)D serum concentration between age categories (2–5 years, 6–11 years, 12–18 years), sex, and season of blood collection were assessed using the Kruskal–Wallis test or Mann–Whitney U test. Post hoc analysis was performed using nonparametric pairwise multiple-comparison procedures such as Dunn’s test.

Multinomial logistic regression was used to test and quantify impact of age category, sex, and season type on odds of VDD and VDI, respectively. The effect size was expressed as an odds ratio or adjusted odds ratio with a 95% confidence interval.

All statistical analysis was performed in R software, version 4.3.2 [33].

## 3. Results

### 3.1. Description of the Studied Sample

Table 1 summarizes distributions of demographic characteristics and serum levels of 25(OH)D in the studied sample. The sex distribution shows that 46% of the pediatric patients were female, while 54% were male, with a male/female sex ratio of 1 to 1. In terms of the serum 25(OH)D status, most of the sample (60%) fell into the “insufficient” or “deficient” status (Table 1).

### 3.2. Distributions of Serum 25(OH)D Values in Pediatric Patients Aged 2–18 Years, Stratified by Age, Sex, and Season of Blood Sample Collection

We found a significant difference in the serum 25(OH)D values in male and female patients (Mann–Whitney U test, *p* = 0.02), showing that male patients had a significantly higher median value than females (difference in medians, 95% CI: 1.7 (0.3, 2.9)).

Overall, we found a statistically significant difference in the distribution values of 25(OH)D among age categories (Kruskal–Wallis test, *p* < 0.001). The post hoc analysis presented in Table 2 showed that children aged 2–5 years had a significantly higher median values than children aged 6–11 years (difference in medians, 95% CI: 11.6 (9.8, 13.3)) and children aged 12–18 years (difference in medians, 95% CI: 13.4 (12.0, 14.9)). We also found that children aged 6–11 years (difference in medians, 95% CI: 11.6 (9.8, 13.3)) had significantly higher values than children aged 12–18 years (difference in medians, 95% CI: 1.8 (0.7, 3.0)).

The comparison of serum 25(OH)D levels across seasons revealed that the highest median concentration was observed during summer (28.9 ng/mL, 95% CI: (27.8, 31.6)), followed by winter (25.6 ng/mL, 95% CI: (24.5, 26.8)), and spring (25.3 ng/mL, 95% CI: (24.4, 26.23)). However, despite these seasonal variations, all recorded median values for each season were within the 25(OH) vitamin D status considered “insufficient” (Table 2).

Table 3 highlights the 25(OH)D status of the sample and its association with age categories, sex, and the season type for blood sample collection. We noticed that in the age group of 2–5-year-olds, many children had a sufficient level of vitamin D (410 (68%)), while in children aged 6–11 years old and 12–18 years old, many of them had insufficient or deficient levels of 25(OH)vitamin D (432 (71%) and 373 (82%), respectively). The frequency of VDI or VDD was significantly higher for children aged 6–11 years (71%, 95% CI: (70, 74)) and children aged 12–18 years (82%, 95% CI: (78, 86)) compared to children aged 2–5 years (32%, 95% CI: (27, 34)). We also observed a significantly higher frequency of VDD among females compared to males (31%, 95% CI: (27, 34) vs. 24%, 95% CI: (21, 26)).

In Table 4, we tested the associations between 25(OH) vitamin D status and age category or sex within the three studied seasons. The association between age categories and 25(OH) vitamin D status remained significant during each of the three studied seasons (*p* < 0.05). In contrast, the association between vitamin D status and sex did not remain significant in spring (*p* = 0.08) and summer (*p* = 0.56).

The results of multinomial logistic regression showed that the age category, sex, and season of blood collection were significantly associated with odds of deficit status of 25(OH) vitamin D (Table 5). After adjusting for season of blood collection, a higher odd of a deficit status of 25(OH) vitamin D was found among children aged 6–11 years old (adjusted OR = 7, 95% CI: (4.9, 9.4)) and children aged 12–18 years old (adjusted OR = 14, 95% CI: (9.3, 19.6)) relative to those ages 2–5 years old.

## 4. Discussion

The serum level of vitamin D is considered an essential determinant of children’s health status. Vitamin D is obtained from food or synthesized in the skin under the action of ultraviolet rays from the sun and is metabolized in the liver and kidneys into 1,25(OH)2D, which plays a direct role in the transcription of genes related to mineral bone metabolism. Deficiency of 25(OH)D is closely associated with skeletal deformities in children but also with other acute and chronic extraskeletal pathologies [3,22,34,35]. For example, low levels of 25(OH)D have been associated with common childhood infectious diseases, including otitis media, pneumonia, influenza, and urinary tract infections. In addition, the occurrence of respiratory infections, asthma, food allergies, and features of metabolic syndrome in adolescents has also been associated with 25(OH)D deficiency [36]. In this regard, observational studies have indicated that a 25(OH)D blood concentration of 50 nmol/L (20 ng/mL) is necessary to prevent rickets, and 75 nmol/L (30 ng/mL) is needed to maintain overall health in the pediatric population [35].

Nevertheless, it is challenging to ensure the recommended intake of vitamin D from diet alone, as the amount of vitamin D in natural food sources is relatively small [5]. Foods of animal origin, such as fish, meat, offal, eggs, and cheese, are the primary sources of naturally occurring vitamin D [37]. In fact, studies assessing plasma 25(OH)D concentrations in individuals with different dietary regimes have shown that serum vitamin D levels were higher in meat and fish consumers than in vegetarians and vegans [38]. Furthermore, dietary sources of vitamin D include fortified foods, including fortified milk, butter, margarine, breakfast cereals, and plant-based beverages [39]. However, there are global variations in vitamin D intake due to differences in the availability of foods rich in vitamin D, being higher in countries with a high income. Variable food fortification practices around the world, from nonexistent to mandatory, may also affect the food supply of vitamin D [40]. Therefore, vitamin D dietary supplements and adequate sun exposure are usually recommended. Indeed, the primary source of vitamin D3 is the synthesis of 7-dehydrocholesterol (previtamin D3) in the skin under the action of ultraviolet (UVB) rays. The intensity of solar UVB rays varies by season, being higher in summer and weaker in winter [41,42]. Nevertheless, an interesting phenomenon occurs in winter, when 25(OH)D stored in muscles is returned to the serum to maintain 25(OH)D concentrations. More precisely, there is evidence that circulating 25(OH)D accumulates in skeletal muscle cells, which have demonstrated the ability to incorporate vitamin D-binding protein from the blood into the cytoplasm, where it binds to cytoplasmic actin and represents a functional depot of 25(OH)D during the winter months [43,44].

Furthermore, the intensity of solar UVB rays also varies by geographical latitude, increasing as it gets closer to the equator. The decreased solar intensity and cold temperatures that characterize moderate to high latitudes discourage cutaneous vitamin D synthesis. From October to March, the skin synthesis of previtamin D3 is not detectable above 50° geographical latitude [41,42,45].

When focusing on vitamin D status in populations of children, studies conducted in Europe showed that countries with relatively middle latitudes had a higher prevalence of VDD (5–20%) than southern countries, where the prevalence ranged from 4.2 to 6.9% [46,47].

In the current study, we assessed the prevalence of VDD and VDI in a sample of children admitted to a pediatric hospital in the northwestern region of Romania. The results showed that more than half of the sample had an inadequate vitamin D status. Approximately one-quarter of the entire sample fell into the deficient category regarding vitamin D status, and about one-third fell into the insufficient category, respectively.

In our studied sample, female pediatric patients had a significantly higher frequency of VDD compared to male pediatric patients. Similarly, reports from Romania, the UK, Finland, Republic of Korea, Saudi Arabia, India, and South Africa found lower levels of 25(OH)D in girls than in boys [6,12,23,48,49]. In particular, a study conducted by Peptine and colleagues on a large sample of Romanian patients aged between 0 and 17 years revealed that cases of VDI and VDD were more common in girls than in boys [28]. Likewise, a study conducted by Benameur et al. [7] among pediatric patients attending a healthcare center in Saudi Arabia identified a higher prevalence of VDD in girls than in boys. Elvia et al., 2022 [50], also found girls to be more deficient in vitamin D than boys when assessing vitamin D status in children aged 1 to 17 years attending the outpatient clinics of a hospital in India.

Similarly, in Canadian children aged 2 to 16 years, a greater prevalence of VDD was reported in boys compared to girls. According to this report, 69% of boys and 35% of girls aged 9 to 16 years had VDD, while in children aged 2 to 8 years, this reached 22% and 8%, respectively. Numerous researchers note that the prevalence of VDD in childhood and adolescence rises with age [47,51].

Our study showed that in children aged 2 to 5 years, the median values of vitamin D were significantly higher than in children aged 6 to 11 years and 12 to 18 years, and that children aged 6 to 11 years had significantly higher values than children aged 12 to 18 years. In a similar manner, a study conducted in Belgium on children aged 0–17 years who underwent a blood test, including a vitamin D level, in a hospital network showed that VDD/VDI had a high prevalence, especially in children above 7 years of age [5].

In our study, in the age group 2–5 years, the level of serum 25(OH)D categorized as deficient was 11%, and that of insufficient was 22%. Similar trends were found in a study from Saudi Arabia, where young children (2–5 years) had lower levels of VDD compared to higher age categories [7]. In New Zealand, the prevalence of VDD in preschool children is reported at 7%, referring to the country’s more southern latitude [47,52].

By comparing serum 25(OH)D levels across seasons, our study revealed that the highest median concentration was observed during summer, followed by winter and spring. However, despite seasonal variations, all median values recorded for each season fell into insufficient vitamin D status.

In a study conducted by Chlebna-Sokół and colleagues, a representative sample of children from various regions of Poland, aged 9 to 13 years, was included. The results indicated that the prevalence of VDD after the winter period reached 84.2% throughout Poland, while the prevalence after the summer period was 26% [11,32,53].

Studies conducted in Australia mention weather conditions, especially the season, as an independent predictor of VDD, with a higher prevalence in the cold than in the warm season [20,21,22].

Yang and colleagues showed that the deficiency and insufficiency of vitamin D in hospitalized children in mainland China were 22.61%, with lower concentrations in girls and children visiting the hospital during winter [54,55]. In another study from China, the prevalence of VDD (<20 ng/mL) was 48.1% in the preschool group (3–6 years) and 21.2% in the young children group (1–3 years). It is also mentioned that in this study, no difference was observed between the sexes [2,56].

In our sample, the level of 25(OH)D and its association with age, sex, and season were observed, showing that in the age group 2–5 years, 61% had a sufficient level of vitamin D. In comparison, in the age groups 6 to 11 years and 12 to 18 years, children had insufficient or deficient levels of vitamin D. Children aged 6 to 11 years and 12 to 18 years had significantly higher serum 25(OH)D levels categorized as deficient and insufficient than the age group 2 to 5 years.

Similar to our study, recent data in the literature have reported high variations in VDD, with the highest prevalence recorded in the eastern Mediterranean region, for Iraq (31.1%), Saudi Arabia (37.4%), and Kuwait, where a prevalence of 58.9% of VDD was recorded in school-aged children [7,57,58].

Studies show that European adolescents aged 15 to 18 years had a higher prevalence of VDD (between 12.2 and 39.6%) than other age groups (between 0.9 and 19.6%), a phenomenon observed previously in other studies [17,59,60,61]. The prevalence of VDD among adolescents in Australia is mentioned at 17%. Among the factors influencing this deficiency, it is discussed that the prevalence of VDD increases with latitude, with lower prevalence in areas closer to the equator and higher prevalence in more distant areas [21,22].

Studies evaluating serum vitamin D levels in Korean adolescents demonstrated that 54.7% of adolescents had VDD, while 13.4% had severe deficiency. Also, these studies reported the lowest levels of 25(OH)D after the winter period and in early spring [62,63,64].

This discrepancy may result in adolescents, partly from increased vitamin D requirements due to pubertal growth and rapid skeletal mass accumulation. Other researchers have also reported this finding, which is mentioned for most European and American populations during growth [32,53,65,66].

A study evaluating VDD in children aged 10 to 18 years from Sri Lanka, a tropical country with year-round sun exposure, found that 13.2% of children aged 10 to 18 years had VDD and 45.6% had VDI, respectively [6,19,47].

Finally, our study has the great strength that it contributes to filling the knowledge gap regarding vitamin D status in the pediatric population of Romania. However, this study may also have some limitations. First, a limitation of our study may be that children aged 0–2 years were not considered in our research, and the autumn season was also not included. Secondly, due to its observational retrospective nature, the study has no information available regarding the patterns of vitamin D supplementation, vitamin D dietary intake, and sun exposure in the studied sample. Moreover, the indication for the blood analysis was not available in the laboratory records analyzed in the framework of the present research, and this potential bias should be taken into account when interpreting the results. Additionally, children admitted to a hospital were selected for the study population. Therefore, the sample may not be perfectly representative of the general population. However, it is worth noting the large size of the study sample (n **=** 1674) and the fact that this sample did not include children with underlying chronic diseases, such as absorption disorders, genetic syndromes, or skeletal malformations. Furthermore, doctors requesting laboratory examinations might have had a clinical suspicion of VDD, which was not taken into consideration in our investigation, and which could generate an additional bias. Lastly, due to the lack of data regarding the diagnoses of pediatric patients included in our study, conclusions about the relationship between vitamin D deficiency and health status cannot be drawn.

Therefore, further research is required to understand the meaning of our findings. It is of interest to assess the relationship between vitamin D status in children and patterns of sun exposure, vitamin D dietary intake, and vitamin D supplementation and to analyze the relationship between vitamin D status and the presence of some pathologies in pediatric patients. Identifying the particularities of these relationships could be used to prevent and treat vitamin D deficiency and to control diseases related to this deficiency more effectively. Also, future studies are required to assess vitamin D status and its determinants in Romanian children aged 0 to 2 years. This category of the pediatric population needs to be studied separately in the context where vitamin D status may present specificities at this young age due to vitamin D supplementation being a more frequent practice in Romanian children 0–1 years old [24]. Indeed, a previous study conducted on a large sample of Romanian patients aged 0 to 85 years showed that children aged 0–2 presented the highest percentage of vitamin D sufficiency [24].

## 5. Conclusions

The number of studies assessing vitamin D status in different populations worldwide has increased recently, with evaluations conducted by measuring serum concentrations of 25(OH)D. The present study revealed variations in the vitamin D status of 2- to 18-year-old pediatric patients according to age, sex, and season. In particular, the study showed a high prevalence of vitamin D deficiency and insufficiency among school-aged children (6–11 years) and adolescents (12–18 years), especially in females and during winter. These findings highlight the importance of monitoring vitamin D levels, especially in seasons when serum 25(OH) vitamin D levels tend to be lower, to mitigate the impact of vitamin D deficiency, particularly among a population in its growth period.

Improving vitamin D status can be achieved by raising awareness of the prevalence of vitamin D deficiency and the importance of adequate sun exposure according to the season, consuming foods rich in vitamin D, and judicious supplementation with vitamin D.

## Figures and Tables

**Table 1:** Demographic characteristics in the studied sample of pediatric patients aged 2–18 years.

Characteristics	All Sample (n = 1674)
Age (years) ^(a)^	8 (5)
Age category (years) ^(b)^	
2 to 5	606 (36)
6 to 11	613 (37)
12 to 18	455 (27)
Sex ^(b)^	
Male	908 (54)
Female	766 (46)
Season of blood sample collection ^(b)^	
Spring	957 (57)
Summer	151 (9)
Winter	566 (34)
25(OH)D Serum concentration (ng/mL) ^(c)^	26 (20; 36)
Vitamin D status ^(b)^	
Sufficient	673 (40)
Insufficient	553 (33)
Deficient	448 (27)

25(OH)D: 25-hydroxyvitamin D; data presented as ^(a)^ mean (standard deviation); ^(b)^ n = number of subjects; ^(c)^ median (25th percentile, 75th percentile); estimated interval for IQR is the inclusive interval.

**Table 2:** Serum 25(OH)D values in the pediatric patients aged 2–18 years stratified by age category, sex, and season types.

Variables	Level of Measure	25(OH)D Serum Concentration (ng/mL)			
		Median (IQR)	Mean (SD)	*p*-Value	Adjusted *p*-Value for Pairwise Comparisons
Age category (years)	2–5	35 (26, 47)	37 (14)	<0.0001 *	<0.0001 *^(a)^
	6–11	24 (18, 31)	25.91 (11)		<0.0001 *^(b)^
	12–18	22 (18, 27)	23.35 (9)		0.0001 *^(c)^
Sex	Male	27 (21, 36)	29.61 (13)	0.02 *	NA
	Female	25 (19, 35)	28.76 (13)		
Season of blood sample collection	Spring	25 (19, 35)	28.81 (13)	0.0003 *	0.0002 *^(d)^
	Summer	29 (24, 39)	32.58 (13)		0.59 ^(e)^
	Winter	26 (20, 36)	28.99 (13)		0.0006 *^(f)^

25(OH)D: 25-hydroxyvitamin D; IQR = (25th percentile, 75th percentile); all intervals estimated for IQR are the inclusive intervals; *p*-values obtained from Mann–Whitney test or Kruskal–Wallis test; * significant result: *p*-value < 0.05; ^(a)^ adjusted significance obtained from Dunn’s test for the comparison between children aged 2–5 years and children aged 6–11 years; ^(b)^ adjusted significance for the comparison between children aged 6–11 years and children aged 12–18 years; ^(c)^ adjusted significance for the comparison between children aged 2–5 years and children aged 12–18 years; NA = not applicable; ^(d)^ adjusted *p*-values obtained from Dunn’s test for the comparison between spring and summer seasonal variations; ^(e)^ adjusted significance for the comparison between spring and winter seasonal variations; ^(f)^ adjusted significance for the comparison between summer and winter seasonal variations.

**Table 3:** Associations between 25(OH)D status and age category, sex, and type of season.

Variables	Level of Measure	25-(OH)D Status			
		Sufficient (≥30 ng/mL)	Insufficient (21–29 ng/mL)	Deficient (<20 ng/mL)	*p*-Value
Age category (years)	2–5	410 (68)	130 (22)	66 (11)	<0.0001 *
	6–11	181 (30)	229 (38)	203 (33)	
	12–18	82 (18)	194 (43)	179 (39)	
Sex	Female	290 (38)	242 (32)	234 (31)	0.006 *
	Male	383 (42)	311 (34)	214 (24)	
Season of blood sample collection	Spring	371 (39)	313 (33)	273 (29)	0.003 *
	Summer	74 (49)	57 (38)	20 (13)	
	Winter	228 (40)	183 (32)	155 (27)	

25(OH)D: 25-hydroxyvitamin D; *p*-values obtained from Chi-squared tests; * significant result: *p*-value < 0.05

**Table 4:** Associations between 25(OH)D status and age category or sex stratified by seasonal variations.

Season	Age Category (Years)		25(OH)D Status		
		Sufficient (≥30 ng/mL)	Insufficient (21–29 ng/mL)	Deficient (<20 ng/mL)	*p*-Value
Spring	2–5	222 (65)	72 (21)	46 (14)	<0.0001 *
	6–11	101 (29)	132 (37)	121 (34)	
	12–18	48 (18)	109 (42)	106 (40)	
Summer	2–5	54 (80)	12 (18)	2 (3)	<0.0001 *
	6–11	14 (32)	22 (50)	8 (18)	
	12–18	6 (16)	23 (59)	10 (26)	
Winter	2–5	134 (68)	46 (23)	18 (9)	<0.0001 *
	6–11	66 (31)	75 (35)	74 (34)	
	12–18	28 (18)	62 (41)	63 (41)	
Season	Sex				
Spring	Female	170 (38)	136 (30)	143 (32)	0.08
	Male	201 (40)	177 (35)	130 (26)	
Summer	Female	31 (44)	29 (42)	10 (14)	0.56
	Male	43 (43)	28 (35)	10 (12)	
Winter	Female	89 (36)	77 (31)	81 (33)	0.03 *
	Male	139 (44)	106 (33)	74 (23)	

25(OH)D: 25-hydroxyvitamin D; *p*-values obtained from Chi-squared tests; the percentage were calculated by rows; * significant result: *p*-value < 0.05.

**Table 5:** Odds ratios and confidence intervals for insufficient or deficient levels of 25(OH)D in pediatric patients stratified by season of blood sample collection.

	25-(OH)D Status			
Factors	Insufficient (21–29 ng/mL)		Deficient (<20 ng/mL)	
	OR (95% CI)	Adjusted OR (95% CI)	OR (95% CI)	Adjusted OR (95% CI)
Age category (years)				
2–5	Reference	Reference	Reference	Reference
6–11	4 (3.0, 5.3)	4 (3.0, 5.3)	8 (5.0, 9.7)	7 (4.9, 9.4)
12–18	7 (5.4, 10.3)	7 (5.4, 10.3)	14 (9.4, 19.6)	14 (9.3, 19.6)
Sex				
Male	Reference	Reference	Reference	Reference
Female	0.97 (0.82, 1.29)	1.01 (0.82, 1.29)	1.44 (1.14, 1.84)	1.43 (1.10, 1.86)
Season of blood sample collection				
Summer	Reference	Reference	Reference	Reference
Spring	1.10 (0.75, 1.60)	1.03 (0.68, 1.55)	2.7 (1.6, 4.6)	2.5 (1.5, 4.4)
Winter	1.04 (0.70, 1.55)	0.96 (.62, 1.47)	2.5 (1.5, 4.3)	2.3 (1.3, 4.1)

25(OH)D: 25-hydroxy vitamin D; 95% CI = 95% confidence interval; all estimated 95% confidence intervals are inclusive intervals.

## Data Availability

The data presented in this study are available on request from the corresponding author.
